# Efficient near infrared light emitting electrochemical cell (NIR-LEEC) based on new binuclear ruthenium phenanthroimidazole exhibiting desired charge carrier dynamics

**DOI:** 10.1038/s41598-017-16133-7

**Published:** 2017-11-16

**Authors:** Babak Nemati Bideh, Hashem Shahroosvand

**Affiliations:** Chemistry Department, University of Zanjan, Zanjan, Iran

## Abstract

Near-infrared light-emitting electrochemical cell (NIR-LEEC) has emerged as a new and promising lighting sourcewhich could serve as low-cost alternatives in NIR light-emitting sources which are typically expensive. LECs were also shown advantages such as light weight, simplicity and low operation voltages. However, only a few examples of NIR-LEEC are reported in which external quantum efficiency(EQE) of devices limited to 0.1%. Here, we report, efficient NIR-LEEC based of two novel binuclear ruthenium phenanthroimidzole complex which differ by employing the type of ancillary ligand including 2, 2′bipyridine (bpy) (B1) and 4, 4′ dimethyl bpy (B2) that realize maximum EQE of 0.14 and 0.68% and extremely long excited state lifetimes of 220 and 374 ns for thin film were estimated, respectively, indicating that influences of substitution on ancillary ligand. Moreover, this substitution dramatically influences other electroluminescence metrics including decreasing turn on voltage from 4.5 to 3.1 V, increasing maximum luminance (L_max_) from 193to 742 cd.m^−2^ and increasing lifetime from 539 to 1104 second, which are the best value among the binuclear ruthenium polypyridyl complexes to date.

## Introduction

Light Emitting Electrochemical Cell (LEEC) has attracted great interest among scientists due to its outstanding photophysical properties^1–3^. In fact, a big challenge that always limits the commercialization of Light Emitting Diodes (LEDs) including multi-layer depositions by deposited stepwise which in most case needs to thermal vacuum evaporation and then rigorous encapsulation process for fabrication of low work function metal or a chemically n-doped electron injection layer which both are unstable in air. These limitations can be resolved through a very simple architecture and low-cost of a LEC^4–8^. In contrast to other types of LED including semiconductor-LED or organic light emitting diode (OLED), a LEC constructs only single layer emitter sandwiched between anode and cathode^9^. The presence of single active layer increases the rate of combination of hole and electron within the emitters when can eventually bring down the turn on voltage below 4 V^10^. Important modification of LEEC occurs upon designing new emitters that can be compatible with electrodes^11^. Up to now, many molecules including transition metal complexes^12^, polymers, Organic- inorganic composites and macromolecules^13^ have been applied in LEEC as emitter. However, the unique photophysical properties of ionic transition metal complexes (ITMCs) such as active triplet excited state, long life time and high quantum efficiency in Ir-cyclometalted complexes and ruthenium polypyridyl complexes caused to design their novel derivatives^14^. In 1996, the first LEEC based on ITMC was reported by Lee and co-workers who indicated that ionic ruthenium polypyridyl complexes have acceptable electroluminescence characteristics and thermal stabilities^15^. A. J. Bard significantly progressed LEC based on [Ru(bpy)_3_]^2+^ (bpy: 2,2-bipyridine) and its analogues which shown high external quantum efficiency (EQE) and luminescence quantum yields (Φ) of about 10%^16^. In 2006, Bolink group suggested a blended of cationic ruthenium tris bathophenthroline complex with 20% poly(methyl methacrylate) (PMMA) which shown a maximum light output of 390 cd/m^2^ at a very low applied voltage (3 V)^17^. Among many factors that affect the electroluminescence (EL) characteristics, the extension of pi-conjugate and atom substitution on the ligands will be able to produce required properties. For instant, pi-conjugation of ligand influence on the HOMO-LUMO band gap to produce desirable electroluminescence color emission^18^. The NIR luminescence material attracts great interest because of prominent applications such as bio-imaging^19–22^, telecommunications^23,24^, and wound healing^25,26^, However, the external quantum efficiency (EQE) value in electroluminescence emitting devices is lower than 0.1% due to the intrinsic difficulty of energy gap law that disfavors radiative transition at higher emission wavelength, indicating the difficulty to obtain the high EQE NIR which made the literature is still lacking^4–7^. Binuclear complexes offer interesting photophysical properties, such as switchable response between two metal core and overlap of emission properties to improve the emission properties of their mono-nuclear complexes^27^.

**Figure 1:**
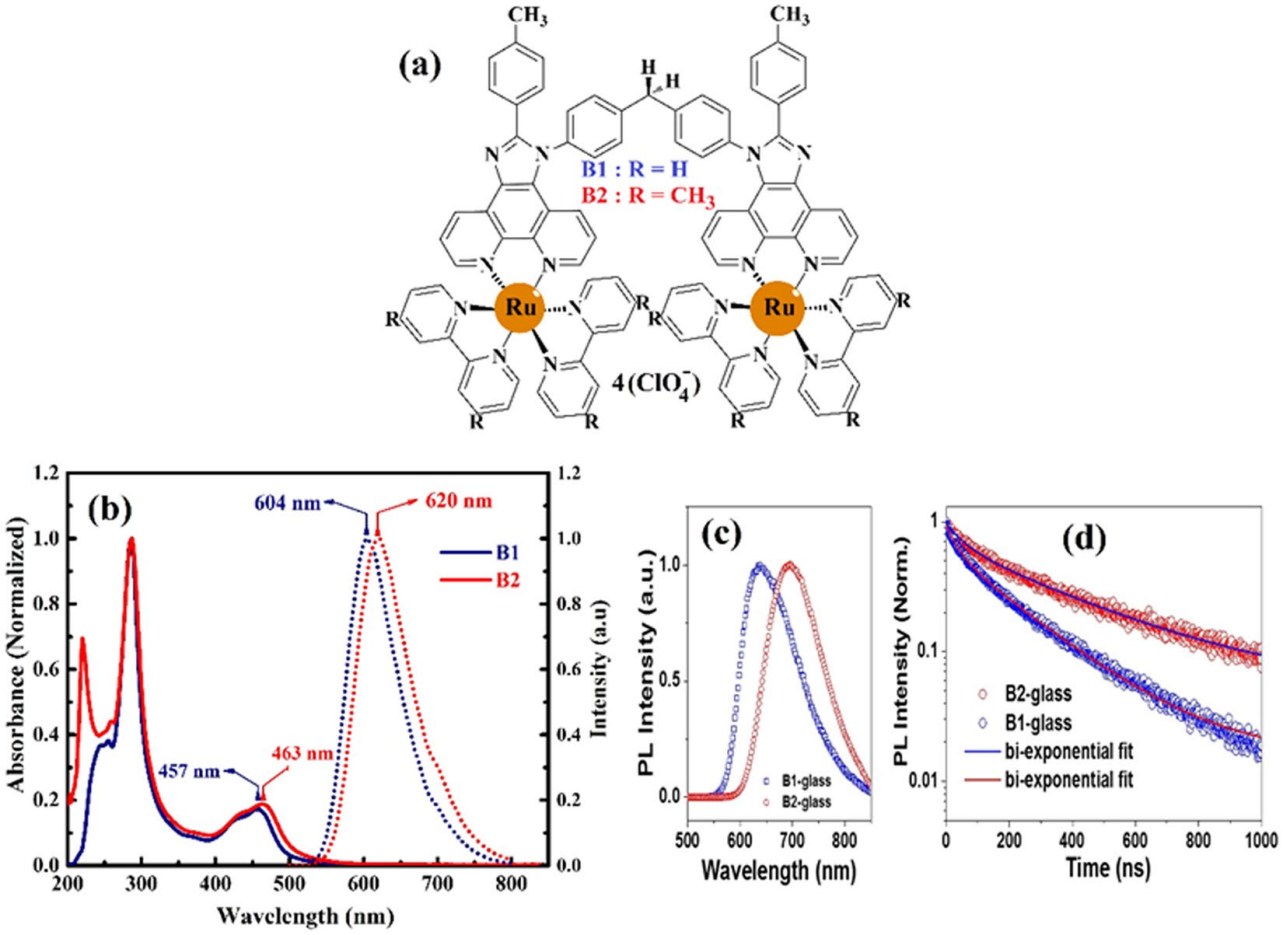
(**a**) Chemical structures of binuclear ruthenium(II) complexes, (**b**) UV-Vis absorption spectra and emission spectra of binuclear complexes in acetonitrile solution,(**c**) Steady-state photoluminescence recorded upon excitation at 450 nm on non-conducting glass substrate (**d**) photoluminescence decay kinetics (PLDK) measured at λ_max_ upon excitation at 408 nm on non-conducting glass substrate.

As part of our project under entitle “the influence of phenanthoimidazole ligand substitution on electroluminescence properties to reach an efficient NIR-LEC^28^, the phenanthroimidazole mononuclear ruthenium complexes with bipyridine and dimethyl bipyridine as ancillary ligand showed the best results in luminance (2395 cd/m^2^ and 1965 cd/m^2^), driving voltage (2.5 and 2.3 V) and EQE (0.689 and 0.845%) metrics among their analogues to date^28^. However, these devices were suffered from low stability as one of the important feature in LEC devices. To overcome this limitation, the increasing of the number of metal core to produce multi nuclear ruthenium complexes is a promising way to increase the stability of LEC device^29^. Therefore, to increasing the stability of LEC based on phenanthroimidazole ligand, we designed and synthesized two new phenanthroimidazole binuclear ruthenium complexes with bipyridine and dimethyl bipyridine as ancillary ligand. We summed two important factors in designing desired complexes. First, binuclear ruthenium complex based on phenathroimidazole ligand was synthesized and then electron donor group substitution on ligand was investigated. The molecular structure of complexes is shown in Fig. 1a.

## Results

The structure of new ligands and complexes were fully characterized and summarized (ESI, S1). Moreover, the aliphatic hydrogen between two phenanthroimidazole germinal moieties play a key role to indicate the formation of binuclear complexes which can be clearly assigned by ^1^HNMR. Signals at 4.4 and 2.4 ppm can be assigned to aliphatic hydrogens in ligand and complexes which are documents for the formation of binuclear compounds (ESI. S2). All characterization techniques establish the formation of two derivatives of ancillary ligands with 2,2 bipyridyl and 4, 4 dimethyl 2, 2 bipyridyl moieties.

**Figure 2:**
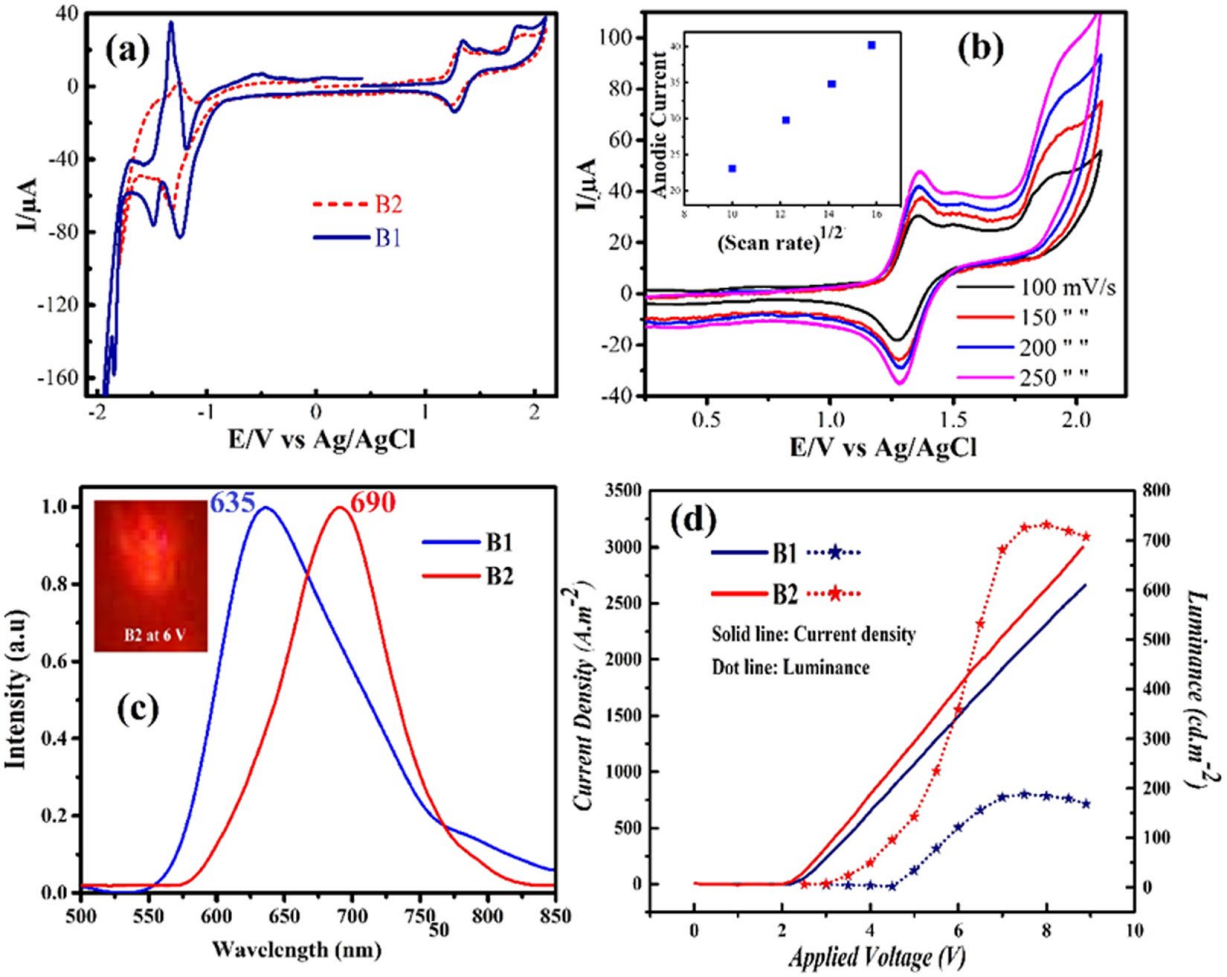
(**a**) Cyclic voltammetry of binuclear ruthenium complexes at scan rate of 80 mV/s, (**b**) Cyclic voltammograms of theof B2 at various scan rates of 100, 150, 200, 250, mV s^−1^. Inset: variation of I_p,a_ (anodic current peak) vs (scan rate)^1/2^, (**c**) Electroluminescence spectra of ITO/B1, B2 complexes/Ga:In devices. Inset: The deep red color of B2 at 6 V, (**d**) Current density and Luminance over applied voltage for ITO/B1, B2 complexes/Ga:In devices.

The Uv-Vis spectra of complexes were recorded in acetonitrile which is shown in Fig. 1b. Two major bands were realized around 200–300 nm and about 450 nm, which can be attributed to *π* → π* transition and metal to ligand charge transfer (MLCT), respectively^30^. The compound B2 with dimethyl derivatives shows a red shift due to the electron donating nature of ancillary ligand. The photoluminescence of compounds in both solution and solid film was examined. In solution, B1 and B2 exhibit emission at 604 and 620 nm, respectively. For the same complexes supported on the non-conductive glass, PL peaks around 645 and 700 nm were observed (Fig. 1c). This difference can be attributed to polarity effects of the medium, as previously reported in the literature^31,32^. Furthermore, the effect of electron donating group on the dynamics of recombination was investigated using time-correlated single photon counting (TCSPC) technique. Generally, the life time of MLCT excited state of ruthenium polypyridyl complexes recorded at room temperature in solution media is in the range of 1–1000 ns^33–40^. However, there is a lacking in the life time data in solid thin film for ruthenium polypyridyl complexes which directly is a key parameter to investigate the performance of every solid - lighting emitting device. Due to the forbidden nature of recombination occurring through MLCT excited state, the emission is long lasting (Fig. 1d). After fitting the PL decay traces with the bi-exponential decay model, we estimated extremely long life time of charge carriers in both films (Table S1). However, as compared to B1 (τ = 220 ns) complex, the recombination was found to be slower in B2 (τ = 374 ns) complex, possibly due to the presence of electron donating group^41,42^. In addition to, B2 complex showed the better lifetime on the glass substrate rather than [Ru(bpy)_3_]^2+^ (τ = 358 ns) as benchmark complex (see Table S5, ESI)^43^. Therefore, we envisage that B2 complex can be a better candidate than B1 complex for the lighting application. The redox properties of complexes were determined by cyclic voltammetry (Figure 1a) and the extracted formal half-wave potential values of the reversible processes are summarized in Table 1. Generally, two regions of oxidation/reduction (Ox/Red) were recognized for ruthenium polypyridyl complexes; the positive and negative regions which can be attributed to Ru(II)→Ru(III) and the Ox/Red of ligands, respectively^44^. That behavior can be seen in our complexes except the second redox wave is in positive region which can be attributed to the oxidation of phenanthroimidazole ligand (ESI. Figure S4). Figure 2b also shows the oxidation wave of B2 at different scan rates from 0 to 250 mV/s. Moreover, as evident from inset of Fig. 2b, the linear correlation between υ½ and the anodic current can be attributed to mass-transport phenomena that controls the kinetics of the overall process and show the reversibilityof this process. In reversible electrochemical process the electron transfer rate is, at all potentials, greater than the rate of mass transport and the peak potential is independent of the applied voltammetric scan rate^45^, therefore, oxidation potential have been used for accurate calculation of energy level of frontier orbital (HOMO, LUMO) of B1 and B2 at the scan rate of 80 mV/s. The calculation of frontier orbitals were shown that stabilization of HOMO and destabilization of LUMO by introduction of methyl group on bipyridine therewithband gap of B2 decrease respect to B1 (Table 1). Furthermore, according to DFT calculations, the presence of the methyl groups on the dmbpy ligands of complex B2 stabilizes the HOMO of complex B2 compared to complexes B1, in addition to existence of methyl group on bipyridine decrease band gap of B2 rather than B1 (ESI, Figure S5).

**Table 1:** UV-Vis, photoluminescence and electrochemical data.

Complexes	Absorbance^a^ ${{\boldsymbol{\lambda }}}_{{\boldsymbol{\max }}}[{\boldsymbol{nm}}]({\boldsymbol{log}}{\boldsymbol{\varepsilon }})$		Emission^b^ ${{\boldsymbol{\lambda }}}_{{\boldsymbol{\max }}}{\boldsymbol{[}}{\boldsymbol{nm}}{\boldsymbol{]}}$		E_1/2_(${\boldsymbol{\triangle }}{\boldsymbol{E}}$) (V)^e^	E_0–0_^f^ eV	E_HOM0_^g^ eV	E_LUMO_^h^ eV	E_gap_^i^ eV
	Ligand Transitions	MLCT	Solution (*φ* ^c^)	Film^d^					
B1	252 (4.04), 285 (4.97)	457 (4.24)	604 (0.116)	645	1.30 (0.060)	2.33	−5.67	−3.34	2.33
B2	220 (4.91), 287 (4.95)	463 (4.23)	620 (0.099)	690	1.27 (0.084)	2.27	−5.64	−3.37	2.27
Ru(bpy)_3_^2+^	245 (4.4), 290 (4.91)	451 (4.17)	607 (0.095)	648	1.29 (0.079)	2.32	−5.66	−3.34	2.32

^a^In acetonitrile(10^−4^ M). ^b^In deaerated CH_3_CN solutionat 298 K. ^c^PLQYs were determined by comparison with [Ru(bpy)_3_]^2+^ ${\boldsymbol{(}}\phi =\,0.095{\boldsymbol{)}}$. ^d^A layer of complexes with thickness of 90 nm were coated on ITO glass. ^e^The E_1/2_ value of compounds was measured in CH_3_CN with 0.1 M TBAClO_4_^−^ vs. Ag/AgCl at scan rate of 80 mV/s. ^f^E_o-o_ was calculated from the intersection of absorption and emission spectra. ^g^E_HOMO_ = −(E_1/2_ (vs. Fc/Fc^+^) + 4.8). ^h^E_LUMO_ = E_HOMO_ + E_0–0_. ^i^E_gap_ = E_HOMO_ − E_LUMO._

The device structure of ITO/complexes (B1 and B2)/Ga:In emits red color electroluminescence as shown in Fig. 2c. We employed the molten way cathode (Ga:In) because the vacuum based deposition technique of metals, such as Al, Ca and Au has certain limitations. Almost 55 nm of red shift in electroluminescence (EL) spectrum was observed for dimethyl-bpy derivate ancillary ligand (B2) extending to NIR region as compared to bpy ancillary ligand, which can be attributed to the electron donating nature of methyl groups^46^. Figure 2d shows the significant different of current density and luminance measurements over applied of two device based on B1 and B2 complexes. As summarized in Table 2, complex B2 indicates the better EL characteristic than B1 which is the highest value reported for the binuclear ruthenium polypyridyl complexes^47^.

**Table 2:** Data of the light emitting device of ITO/[(Ru(N^N)_2_)_2_  (DiP-methane)]^4+^/Ga:In.

Complexes	*λ*_max_[nm]	CIE^a^	FWHM [nm]	J^b^	L_max_^c^	V_on_^d^	t_on_^e^	t_1/2_^f^	E^g^	EQE (%)^h^
B1	635	[0.652, 0.315]	125	1921	193 (7.4 V)	4.5	58	539	0.12	0.141 (7.50 V)
B2	690	[0.628, 0.309]	122	2224	742 (7.7 V)	3.1	87	1104	0.34	0.682 (5.9 V)

^a^CIE(x, y): Commission Internationale de L’Eclairage, ^b^Current density A m^−2^ at 7 V, ^c^Maximum luminance cd .m^−2^ at voltage that noted in parenthesis. ^d^Turn-on voltage V. ^e^Turn-on time (s). ^f^Lifetime (s). ^g^Efficacy (cd A^−1^) at 7 V. ^h^Maximum external quantum efficiency at voltage that noted in parenthesis.

**Figure 3:**
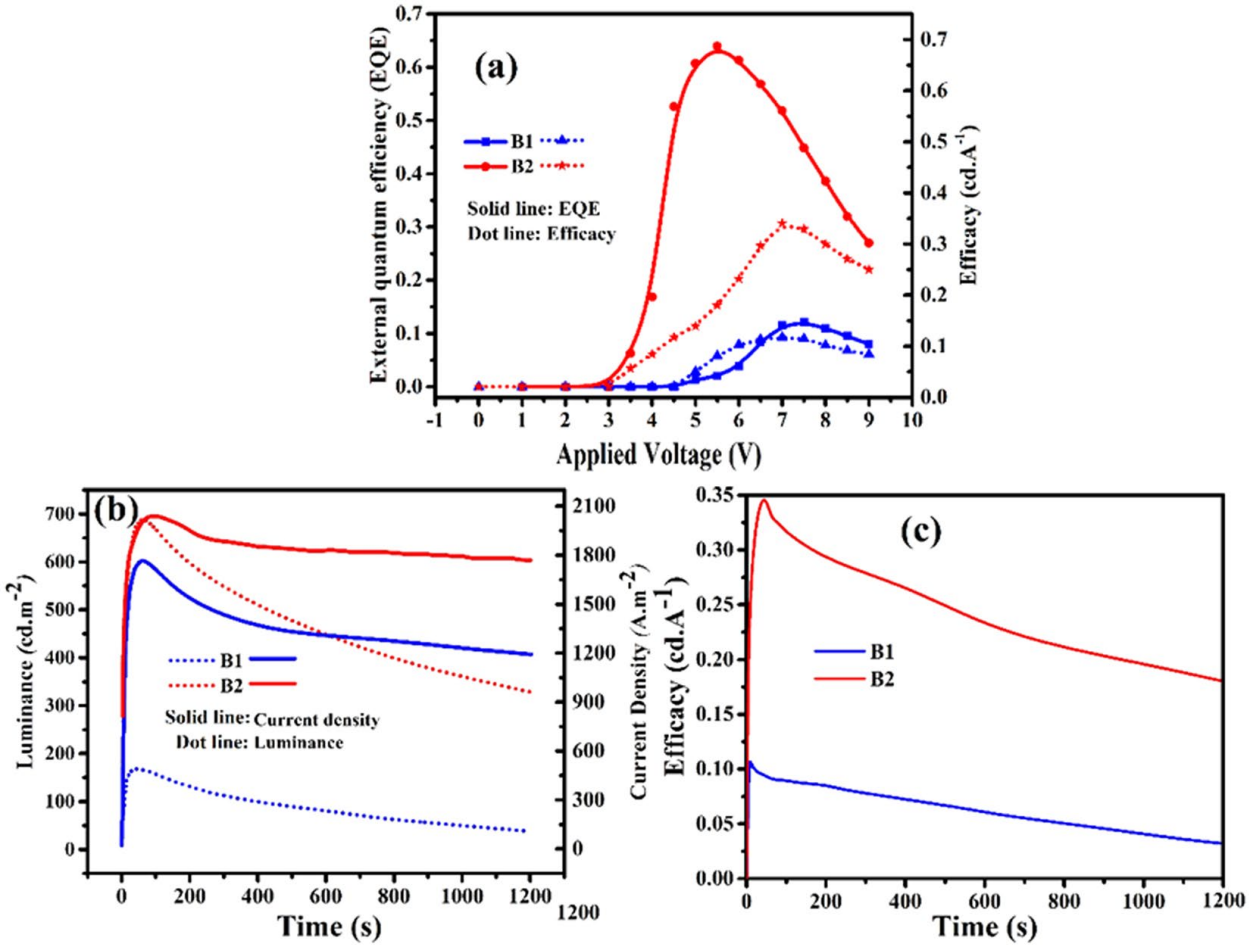
(**a**) External quantum efficiency (EQE) and efficacy (E) as a function of the applied voltage for ITO/B1, B2 complexes/Ga:In devices, (**b**) Luminance and current density evolutionas a function of time at appliedconstant voltage of 7 V for ITO/B1, B2 complexes/Ga:In devices, (**c**) Efficacy over time at applied constant voltage of 7 V for ITO/B1, B2 complexes/Ga:In devices.

Interestingly, the turn on voltage of complexes dramatically reduced from 4.5 V for B1 to 3.1 V for B2. As compared to B1 based device, maximum luminance (L_max_) is about three fold for the device based on B2. Apparently, the substitution of electron donor moieties influences the stability of device performance over time that was more apparent in B2 complex (3b, c). The LEC device based on B1 exhibit the maximum EQE in about 7.5 volt for B1 (0.141%) and about 5.9 volt for B2 (0.682). The device based on B1 show maximum luminance of 194 cd/m^2^ at 7.4 V while based on B2 show 742 cd/m^2^ at 7.7 V. The turn-on time was obtained for B1 and B2, 57 and 87 second, respectively. In addition to we obtained lifetime of 539 s, 1104 sfor B1 and B2, respectively. The lifetimes show the better stability of device based on B2 rather than B1. (Fig. 3 and Table 2).

As our knowledge, the OLED and LEC based on binuclear ruthenium polypyridyl complexes is very rare, which summarized in ESI. Table S4 ^27,29,47–49^. The highest demonstrated EQE value of NIR EL for a binuclear ruthenium polypyridyl complex with an anode ITO/complex (100 nm)/Au (100 nm) configuration is 0.013%^48^, while the EQE value in current work is reached to 0.682 % with the configuration of ITO/B2 (90 nm)/Ga:In.

Although two-layer deposition procedures were completely the same, surprisingly the morphology and arrangement of complexes on the surface is completely different. From SEM images (ESI, Figure S6), we conclude that the methyl group substitution affects the Surface morphology, which eventually improved the characteristics of the EL device.

In comparison with earlier work^28^, dinuclear ruthenium complex (B2) shown a red shift about ∼44 nm to near infrared region compared to its mononuclear analogue (NE02, 630 nm). Later, the device lifetime of B2 was significantly improved compare to its mononuclear analogue (from 720 s for NE02 to 1104 s for B2). It is useful to note that the aim of this work was not to search for the best performances and to maximize the cell efficiencies. On the other hand, the actual goal of this study was to introduce the first NIR-LEC based on binuclear ruthenium phenanthroimidazole and the influence of electron donation group substituent to improve the life time and other EL properties of a NIR-LEC. However, the literature lacks a detailed the solid life time and EL characteristics of binuclear ruthenium polypyridyl complexes and thus, the knowledge of the photopysical and light emitting electroluminescence properties of this very important class is very limited. Therefore, we believe that more reports on binuclear ruthenium polypyridyl complexes are needed to the development of the OLED and LEC based on NIR luminescent materials.

In summary, we successfully tailored the excited state dynamics by molecularly engineering the structure of two new binuclear ruthenium phenanthroimidazole complexes, which were employed as efficient emitters in near infrared light emitting electrochemical cells. Even in the absence of selective contacts, all electroluminescence parameters, including turn on voltage (from 4.5 to 3.1 V) maximum luminance (from 193 to 742 cd m^−2^), lifetime (from 539 s to 1104 s) and EQE (from 0.14 to 0.68 %) improved when emitter containing dimethyl-bpy ancillary ligand instead of bpy ancillary ligand was employed.

## Electronic supplementary material

Supplementary Information

## References

[1] Xu H (2014). Recent progress in metal–organic complexes for optoelectronic applications.. Chem. Soc. Rev..

[2] Costa RD (2012). Luminescent ionic transition-metal complexes for light-emitting electrochemical cells. Angew. Chem. Int. Ed..

[3] Ho C-C (2011). Phosphorescent sensitized fluorescent solid-state near-infrared light-emitting electrochemical cells. Phys. Chem. Chem. Phys..

[4] Xu H, Sun Q, An Z, Wei Y, Liu X (2015). Electroluminescence from europium(III) complexes. Coord. Chem. Rev..

[5] Bolink HJ (2009). Deep-Red-Emitting Electrochemical Cells Based on HeterolepticBis-chelated Ruthenium(II) Complexes. Inorg. Chem..

[6] Hosseini AR (2005). Addition of a Phosphorescent Dopant in Electroluminescent Devices from Ionic Transition Metal Complexes. Chem. Mater..

[7] Pope, M. & Swenberg, C. E. Electronic Processes in Organic Crystals and Polymers, Oxford University Press, New York, 2nd edn, (1999).

[8] Farinola GM, Ragni R (2011). Electroluminescent materials for white organic light emitting diodes. Chem. Soc. Rev..

[9] Meier SB (2013). Dynamic Doping in Planar Ionic Transition Metal Complex-Based Light-Emitting Electrochemical Cells. Adv. Funct. Mater..

[10] Buda MNJ, Paul BJ, Bard A (2003). Stability of thin-film solid-state electroluminescent devices based on tris (2,2′-bipyridine) ruthenium (II) complexes. J. Am. Chem. Soc..

[11] Lin CY, Bard AJ (2002). Individually addressable submicron scale light-emitting devices based on electroluminescence of solid Ru(bpy)3(ClO4)2 films. J. Am. Chem. Soc..

[12] Mak, C. S. K. & Chan, W. K. Electroluminescence from Metal-Containing Polymers and Metal Complexes with Functional Ligands. John Wiley & Sons, Chapter 10, page 329, 2008.

[13] Zhu Y, Fei T, Ma Y (2016). A Highly Efficient red-emitting ruthenium complex with 3,5-difluorophenyl substituents. Chem. Plus. Chem.

[14] Tung Y-L (2005). Organic light-emitting diodes based on charge-neutral Ru (II) phosphorescent emitters. Adv. Mater..

[15] Lee J-K, Yoo DS, Handy ES, Rubner MF (1996). Thin film light emitting devices from an electroluminescent ruthenium complex. Appl. Phys. Lett..

[16] Gao FG, Bard AJ (2002). High-brightness and low-voltage light-emitting devices based on trischelated ruthenium(II) and tris(2,2′-bipyridine) osmium(II) emitter layers and low melting point alloy cathode contacts. Chem. Mater..

[17] Bolink HJ, Cappelli L, Coronado E, Gratzel M, Nazeeruddin MK (2006). Efficient and stable solid-state light-emitting electrochemical cell using tris(4,7-diphenyl-1,10-phenanthroline) ruthenium(II) hexafluorophosphate. J. Am. Chem. Soc..

[18] Tung Y-L (2006). Orange and red orangic light-emitting devices employing neutral Ru (II) emitters: rational design and prospects for color tuning. Adv. Funct. Mater..

[19] Whelan, H. T. *et al*. Effect of NASA Light-Emitting Diode Irradiation on Wound Healing. *J. Clin. Laser Med. Sur*., *19*, 305 (2001). 10.1089/104454701753342758 11776448

[20] Karu, T. The Science of Low Power Laser Therapy, Gordon and Breach Scientific, New York, 1998.

[21] Raghavachari, R. Near-Infrared Applications in Biotechnology, CRC, Boca Raton (2001).

[22] Desurvire, E. Erbium-Doped Fiber Amplifiers: Principles and Applications, Wiley Interscience, New York, 1994.

[23] Slooff LH (2001). Near-infrared electroluminescence of polymer light-emitting diodes doped with a lissamine-sensitized Nd3+ complex. Appl. Phys. Lett..

[24] Curry RJ, Gillin WP (1999). 1.54 μm electroluminescence from erbium (III) tris (8 hydroxyquinoline) (ErQ)-based organic light-emitting diodes. Appl. Phys. Lett..

[25] Bu¨nzli J-CG, Eliseeva SV (2010). Lanthanide NIR luminescence for telecommunications, bioanalyses and solar energy conversion. J. Rare Earths.

[26] Rausch AF, Thompson ME, Yersin H (2009). Blue Light Emitting Ir(III) Compounds for OLEDs -New Insights into Ancillary Ligand Effects on the Emitting Triplet State. J. Phys. Chem. A.

[27] Welter S, Brunner K, Hofstraat JW, De Cola L (2003). Electroluminescent device with reversible switching between red and green emission. Nature.

[28] NematiBideh B, Roldán-Carmona C, Shahroosvand H, Nazeeruddin MK (2016). Ruthenium phenanthroimidazole complexes fornear infrared light-emitting electrochemical cells. J. Mater. Chem. C.

[29] Jia W-L, Hu Y-F, Gao J, Wang S (2006). Linear and star-shaped polynuclear Ru(II) complexes of 2-(2-pyridyl) benzimidazolyl derivatives: syntheses, photophysical properties and red light-emitting devices. Dalton Trans..

[30] NematiBideh B, Roldán-Carmona C, Shahroosvand H, Nazeeruddin MK (2016). Low-voltage, high-brightness and deep-red light-emitting electrochemical cells (LECs) based on new ruthenium (II) phenanthroimidazole complexes. Dalton Trans..

[31] Bolink HJ, Cappelli L, Coronado E, Gavin AP (2005). Observation of Electroluminescence at Room Temperature from a Ruthenium(II) Bis-Terpyridine Complex and Its Use for Preparing Light-Emitting Electrochemical Cells. Inorg. Chem..

[32] Slinker JD (2004). Efficient Yellow Electroluminescence from a Single Layer of a Cyclometalated Iridium Complex. J. Am. Chem. Soc..

[33] Campagna S, Puntoriero F, Nastasi F, Bergamini G, Balzani V (2007). Photochemistry and photophysics of coordination compounds: ruthenium. Top Curr Chem..

[34] Cavazzini, M., Pastorelli, P., Quici, S., Loiseau, F. & Campagna, S. Two-color luminescence from a tetranuclearIr(III)/Ru(II) complex. *Chem Commun* 5266–5268 (2005). 10.1039/b508063k 16244723

[35] Barigelletti, F., Flamigni, L., Collin, J. P. & Sauvage, J. P. Vectorial transfer of electronic energy in rod-like ruthenium–osmium dinuclear complexes. *Chem Commun* 333–338 (1997)

[36] Collin JP, Gavina P, Hietz V, Sauvage JP (1998). Construction of one-dimensional multicomponent molecular arrays: control of electronic and molecular motions. Eur J. Inorg. Chem..

[37] Barigelletti F, Flamigni L (2000). Photoactive molecular wires based on metalcomplexes. Chem. Soc. Rev..

[38] Juris A (1988). Ru(II) polypyridine complexes: photophysics. Photochemistry, eletrochemistry, and chemiluminescence, Coord. Chem. Rev..

[39] Zysman-Colman E, Slinker JD, Parker JB, Malliaras GG, Bernhard S (2008). Improved Turn-On Times of Light-Emitting Electrochemical Cells. Chem. Mater..

[40] Ramachandra S (2011). Luminescent Ruthenium Tripod Complexes: Properties in Solution and on Conductive Surfaces. Inorg. Chem..

[41] Harriman, A. & Ziessel, R. Making photoactive molecular-scale wires. *Chem. Commun.* 1707–1716 (1996).

[42] Benniston AC, Grosshenny V, Harriman A, Ziessel R (1994). Electron delocalization in ethynyl-bridged binuclear ruthenium (II) polypyridine complexes. Angew. Chem. Int. Ed, Engl..

[43] Sciuto EL (2015). Photo-physical characterization of fluorophore Ru(bpy)3^2+^ for optical biosensing applications, Sensing and Bio-Sensing. Research.

[44] Sun Y, Collins SN, Joyce LE, Turro C (2010). Unusual photophysical properties of a ruthenium(II) complex related to [Ru(bpy)2(dppz)]2+. Inorg. Chem..

[45] Bard, A. J. & Faulkner, L. R. Electrochemical Methods: Fundamentals and Applications, 2^nd^ edn (Wiley, New York, 2001).

[46] Gong X, Ng PK, Chan WK (1998). Trifunctional light-emitting molecules based on rhenium and ruthenium bipyridine complexes. Adv. Mater..

[47] Ju C-C, Chen C-H, Yuan C-L, Wang K-Z (2011). Electroluminescence from single-layer thin-film devices based on three binuclear Ru(II) complexes with different length of flexible bridges. Thin Solid Films.

[48] Xun S, Zhang J, Li X, Ma D, Wang ZY (2008). Synthesis and nearinfrared luminescent properties of some ruthenium complexes. Synth. Methods.

[49] Lepretrea J-C, Deronziera A, Stephan O (2002). Light-emitting electrochemical cells based onRuthenium(II) using crown ether as solid electrolyte. Synth. Met..

