# Supplementary material for: Efficient near infrared light emitting electrochemical cell (NIR-LEEC) based on new binuclear ruthenium phenanthroimidazole exhibiting desired charge carrier dynamics
Source: Sci Rep. 2017 Nov 16;7:15739. doi: 10.1038/s41598-017-16133-7 (PMC5691147; doi:10.1038/s41598-017-16133-7)
Supplement: Supplementary file 1 — Supplementary Information [file 41598_2017_16133_MOESM1_ESM.pdf]

**Supplemenatry Information for**

# Efficient near infrared light emitting electrochemical cell (NIR-LEEC) based on new binuclear ruthenium phenanthroimidazole exhibiting desired charge carrier dynamics

Babak Nemati Bideh,[a] Hashem Shahroosvand [a]*

[a]Chemistry Department, University of Zanjan, Zanjan, Iran

S1: Methods and Materials.

All reagents and solvents were purchased from commercialsources and used without further purification.NMR spectra were recorded on a Bruker 250 MHz spectrometer with $CDCl_3$, $D_6$-DMSOand tetramethylsilane (TMS)as solvent and internal reference, respectivelyElemental analyses were performed onElementarVario EL CHN elemental analyzer. IR spectra were recorded on a Perkin-Elmer 597 spectrometer.The Electrochemical studies of ruthenium complexes ($2\times10^{-3}$M) were performed under under a dry $N_2$ atmosphere at 298 K by using SAMA500 potentiostat electrochemical analyzerwith conventional threeelectrode cell, a Pt disk as the working electrode, a Pt wire as the counter electrode, and Ag/AgCl as the reference electrode. The CV measurements were performed at room temperature using 0.10 M tetrabutylammonium perchlorate (TBAP) as the supporting electrolyte and degassed acetonitrile as the solvent. In CV the following parameters and relation were used: scan rate, 30 mV $s^{-1}$; formal potential $E^{o'} = (E_{pa} +/E_{pc})/2$ where $E_{pa}$ and $E_{pc}$ are anodic and cathodic peak potentials, respectively; $\Delta E_p$ is the peak-to-peak separation. The oxidation ($E_{ox}$) and reduction ($E_{red}$) potentials were used to calculate the HOMO/LUMO energy levels and energy gap ($E_{gap}$) using the equations $E_{HOMO}$=-($E_{ox}$(vs.$F_c/F_C^{+}$)+ 4.8 eV), $E_{LUMO}$ = $E_{HOMO}$ + $E_{0\text{-}0}$ eV, $E_{0\text{-}0}$ was calculated from the intersection of absorption and emission spectra in acetonitril solutin. And$E_{gap}$= -$E_{HOMO}$ – $E_{LUMO}$ which is the reduction potential offerrocene which was found to be 0.43 V [1].UV–visible absorption spectra wasrecorded on an Ultrospec3100 pro spectrophotometer in acetonitrile solutions . Photoluminescence (PL) emission spectra of ruthenium complexesin degassed solutionsat 298 K and neat films were made on glass substrates were recorded using Varian-Cary Eclipse flourocence spectrophotometer and AvaSpec-125 spectrophotometer, respectively. Photoluminescence was recorded on a spectrofluorometer Fluorolog 322 by exciting the samples at a fixed wavelength of 450 nm. For the time-resolved photoluminescence studies, the spectrometer working in a time-correlated single-photon counting mode with <ns time resolution was used. Picosecond pulsed diode laser head NanoLED-405LH (Horiba) emitting <200 ps duration pulses at 408 nm with repetition rate of 1 MHz was used as an excitation source.

The PLQY (PL quantum yields) were calculated by comparison with $[Ru(bpy)_3]^{2+}$ in degassed $CH_3CN$ solution at room temperature as a standard ($\emptyset_{std} = 0.095$) [2] using to the well-known following equation:

$$\emptyset_{unk} = \emptyset_{std}.(\frac{I_{unk}/A_{unk}}{A_{std}/I_{std}}).(\frac{\eta_{unk}}{\eta_{std}})^2$$

In equation,$\Phi_{unk}$ is PL quantum yield of ruthenium complexes, $I_{unk}$ and $I_{std}$ are the integrated areas of the corrected PL spectra of the ruthenium complexes and standard respectively, $A_{unk}$ and $A_{std}$ are the absorbances of the ruthenium complexes and the standard at the excitation wavelength ($\lambda_{exc}$= 460 nm), and $\eta_{unk}$ and $\eta_{std}$ are the indexes of refraction of the respective solvents (taken to be equal to the neat solvents in both cases). Thin films of cationic ruthenium complexes for study of solid emission were obtained by drop cast from a spectrophotometric grade acetonitrile solution on a glass support with a thickness of about 90 nm. After evaporation of the solvent in air, the films were dried overnight under vacuum at room temperature.

**S2: Synthesis and characterization:** The compounds cis-[Ru(dmbpy)$_2$Cl$_2$].2H$_2$O, cis-[Ru(bpy)$_2$Cl$_2$].2H$_2$O, cis-[Ru(phen)$_2$Cl$_2$].2H$_2$O, and 1,10-phenanthroline-5,6-dione (phendione) were synthesized according to reference methods [3-6].

The general procedure of synthesis of ligand and complexes is shwon in figure S1. Morover, the $^{1}$HNMR and $^{13}$CNMR of ligand of DiP-methane are shwon in Figures S2 and S3.

**Synthesis of bis(4-(2-(p-tolyl)-1H-imidazo [4,5 f][1,10]phenanthrolin-1-yl)phenyl)methane (DiP-methane):** A mixture of 1,10-phenanthroline-5,6-dione (0.212 g, 1.0 mmol), 4-methylbenzaldehyde (0.120 g, 1.0 mmol), 4,4'-methylenedianiline (0.99 g, 0.50 mmol) and ammonium acetate (770 mg, 10 mmol, excess) was refluxing in glacial acetic acid (15 mL) for 30 h under a inert atmosphere (N$_2$). The reaction mixture was cooled to room temperature, poured into deionized water (45 mL). The suspension mixture treated with a 25% NH$_3$ solution until the pH=6.5 and extracted with 50 mL of chloroform and then removed the solvent by rotary evaporation and the residue was washed with ethanol and acetone. The recrystallization from CH$_2$Cl$_2$–acetone was repeated one more time to give the product as a grey solid. Yield: 58%. mp. 276°C, Anal. calcd. For C$_{53}$H$_{36}$N$_8$ (%): C, 81.10 ; H, 4.62; N, 14.28. Found (%): C, 89.93; H, 4.61; N, 14.29. IR (KBr): $\tilde{\nu}$ = 3074 (C-H aromatic), 2973 (C-H aliphatic), 1619 (C=C), 1605 (C=N), 1371 cm$^{-1}$ .$^{1}$HNMR (250 MHz, CDCl$_3$): 9.15-9.21(m, 4H), 9.03(d, 2H),7.76 (dd, 2H), 7.45-7.61 (m, 12H), 7.21-7.26 (m, 4H), 7.09 (d, 4H), 4.40 (s, 2H), 2.27 (s, 6H). $^{13}$CNMR (62 MHz, CDCl$_3$): 155.21, 152.38, 149.02, 147.81, 144.87, 144.31, 142.49, 139.53, 136.63, 136.18, 130.93, 130.53, 129.15, 129.07, 127.76, 126.95, 126.74, 123.97, 123.55, 121.97, 119.77, 41.21, 21.32.

**General procedure for synthesis of [Ru$_2$(N^N)$_4$(DiP-methane)](ClO$_4$)$_4$:** A mixture of cis-[Ru(N^N)$_2$Cl$_2$].2H$_2$O (0.1 mmol) and DiP-methane (39.2 mg, 0.05 mmol) was degassed by N$_2$ and heated under N$_2$ at 130 °C in ethylene glycol(5 ml) for 24.0 h to give a clear red solution. Upon cooling, the solution was treated with aqueous solution of NaClO$_4$until gave a orange precipitate and washed several times with deionized water to remove traces rest of salts. The crude product was purified by column chromatography on alumina with acetonitril-toluene (3/1, v/v) as an eluent. The mainly red band was collected. The solvent was removed under reduced pressure and red solid were obtained.

**[$Ru_2(bpy)_4$(DiP-methane)]($ClO_4$)$_4$ (B1).** Yield: 73% (79 mg). IR (KBr): $\tilde{\nu}$ = 3082 (C-H aromatic), 2952 (C-H aliphatic), 1623 (C=C), 1598 (C=N), 1527, 1081 ($ClO_4$) $cm^{-1}$. $^1$HNMR (250 MHz, $D_6$-DMSO):9.18 (d, 2H),8.83 (m, 8H), 8.11-8.24 (m, 10H), 7.95 (d, 4H), 7.81 (d, 4H), 7.74 (m, 4H), 7.45-7.68 (m, 20H), 7.35 (m, 4H), 7.09(d, 4H), 4.39 (s, 2H), 2.09 (s, 6H).$^{13}$CNMR (62 MHz, $CDCl_3$):157.09, 157.00, 156.99, 154.30, 151.83, 151.07, 150.14, 145.84, 145.69, 144.35, 140.21, 138.47, 138.31, 136.58, 135.32, 131.58, 131.04, 129.53, 129.45, 128.36, 128.21, 127.61, 127.37, 126.67, 126.00, 125.91, 124.90, 121.91, 21.18. Anal. calcd. For $C_{93}H_{68}Cl_4N_{16}O_{16}Ru_2$ (%): C, 55.583; H, 3.414; N, 11.157. Found (%): C, 55.577; H, 3.404; N, 11.160. ESI-MS: m/z 1811.10, [M-2$ClO_4$]$^{2+}$.

**[$Ru_2(dmbpy)_4$(DiP-methane)]($ClO_4$)$_4$(B2).** Yield: 63% (76 mg). IR (KBr): $\tilde{\nu}$ = 3088 (C-H aromatic), 2946 (C-H aliphatic), 1611 (C=C), 1601 (C=N), 1540, 1089 ($ClO_4$) $cm^{-1}$. $^1$HNMR (250 MHz, $D_6$-DMSO): 9.20 (d, 2H), 8.73 (m, 6H), 8.19 (m, 8H), 7.81 (d, 4H), 7.71 (d, 4H), 7.68 (m, 4H), 7.43-7.61 (m, 18H), 7.43 (d, 4H), 6.99(d, 4H), 4.43 (s, 2H), 2.20 (s, 6H).Anal. calcd. For $C_{101}H_{86}Cl_4N_{16}O_{16}Ru$ (%): C, 57.173; H, 3.992; N, 10.564. Found (%): C, 57.171; H, 3.989; N, 10.561. ESI-MS: m/z 1923.12, [M-2$ClO_4$]$^{2+}$.

**Device fabrication and measurement :** Indium tin oxide (ITO) coated glass with a sheet resistance of 20 Ω/square was used as the transparent anode. After being sufficiently cleaned by soaking in ultrasonicated isopropanol, aceton and deionized water, it was dried in the oven at 110°C for 2h. The devices were prepared by spin-coating a thin layer of each complex (B1, B2) on top of an ITO glass substrate from a 5% (w/v) acetonitrile solution at RT. All solution and film preparation were performed under ambient conditions. The thicknesses of the films were ~ 85 nm, measured with profilometry. After spincoating, the thin films were annealed at 90 °C in inert atmosphere for 14h. A Ga: In (75.5:24.5 wt %, mp 15.7 °C) eutectic as cathode (ca. 3.5 mm diameter) was printed on the top of the active layer at room temperature by using a special syringe (glass syringe with needle diameter about 1.5 mm) [7] and then connected via a thin copper wire inserted into the Ga:In contact. Finally it was sealed with epoxy cement. All EL measurement were carried out in air atmosphere. The current density, luminescence versus the voltage and emission characteristics of LEC devices were measured using an AvaSpec-125 spectrophotometer, a SAMA500 electroanalayzer system and a Photo Research PR-650 spectroradiometer.

Emission lifetime and corresponding intensities for B1 and B2 complex obtained using bi-exponential decay modelare samurized in Table S1. The cyclic voltammetry of ligand in DMF solvent is shown figure S4. Top view SEM images of surfaces of complexes B1 and B2 in fabricated LEC devices is shown in figure S5.

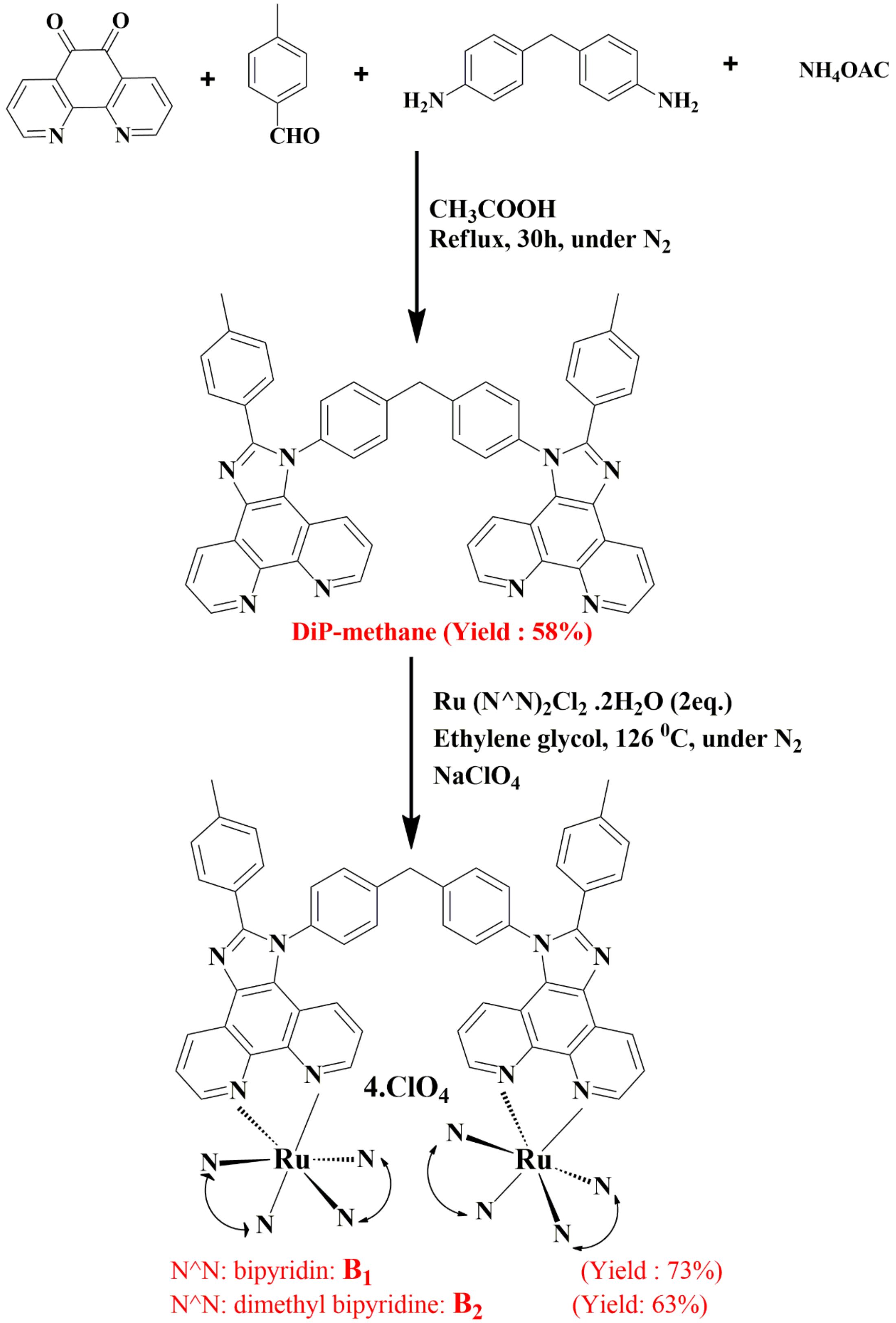


**Figure S1.** Synthesis of phenanthroimidazole ligand (Dip-methane) and their derivative dinuclear ruthenium(II) complexes

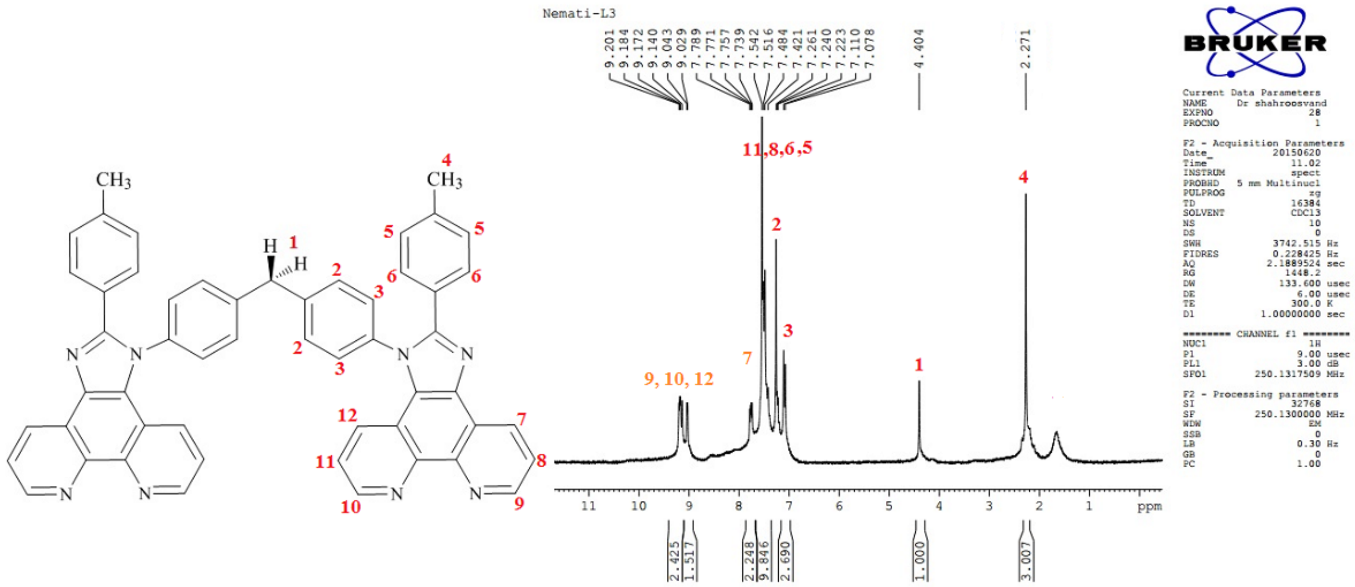


**Figure S2**. $^{1}$HNMR of DiP-methane in $CHCl_3$

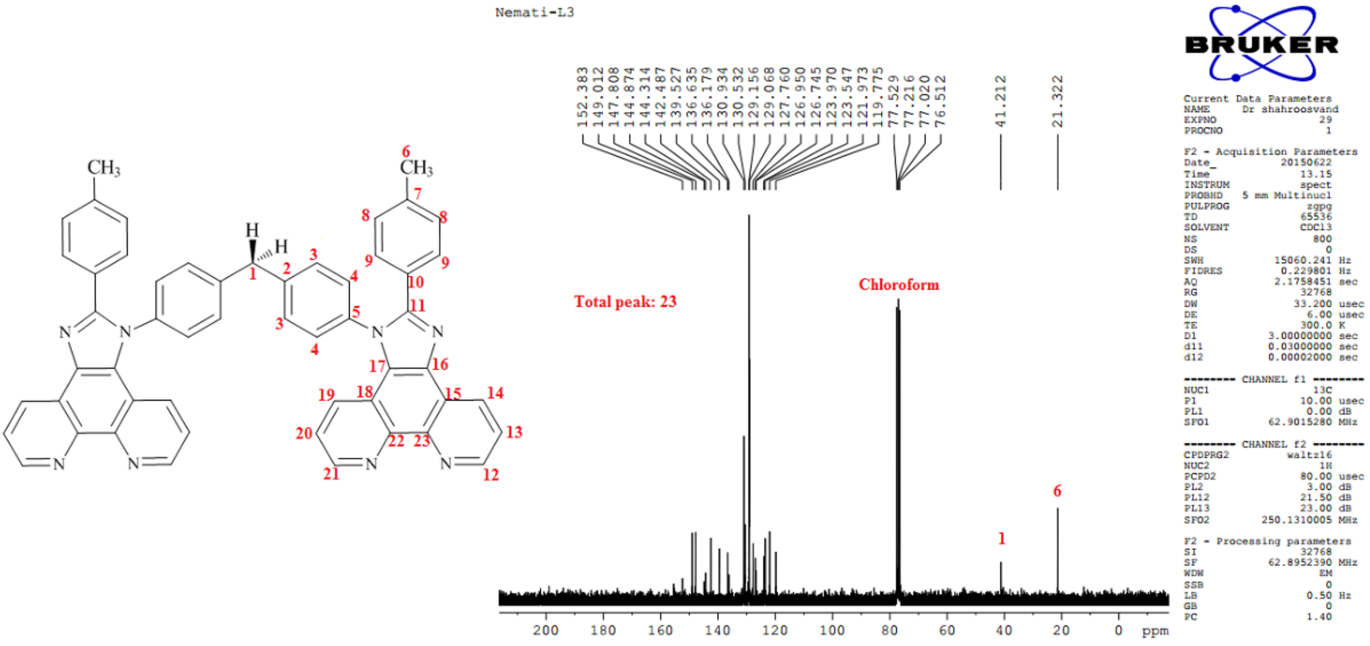


**Figure S3**. $^{13}$CNMR of DiP-methane in $CHCl_3$

**Table S1**. Emission lifetime and corresponding intensities for B1 and B2 complex obtained using bi-exponential decay model.

| Sample | $A_1$ (%) | $\tau_1$ (ns) | $A_2$ | $\tau_2$ (ns) |
|---|---|---|---|---|
| **B1** | 37 | 36 | 63 | 220 |
| **B2** | 30 | 28 | 70 | 374 |

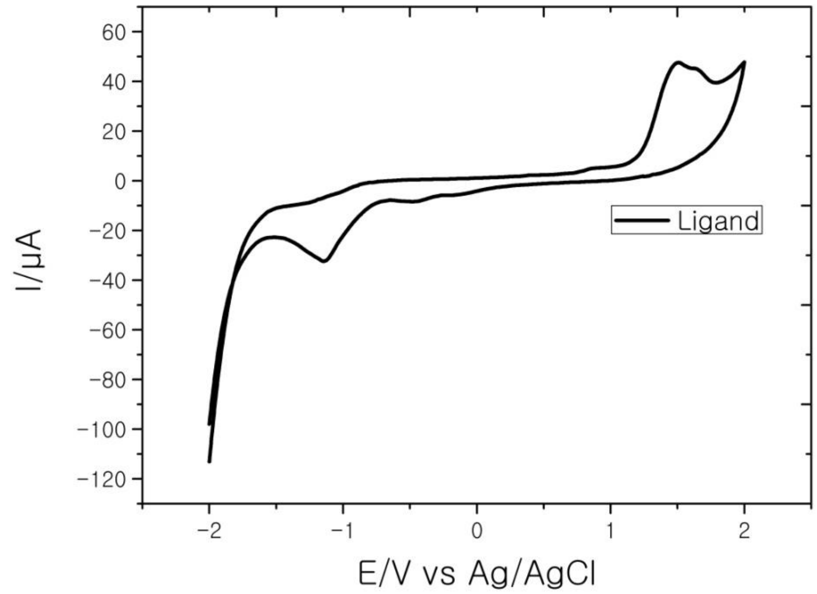


**Figure S4**. The Cyclic Voltammetry of ligand in DMF solvent

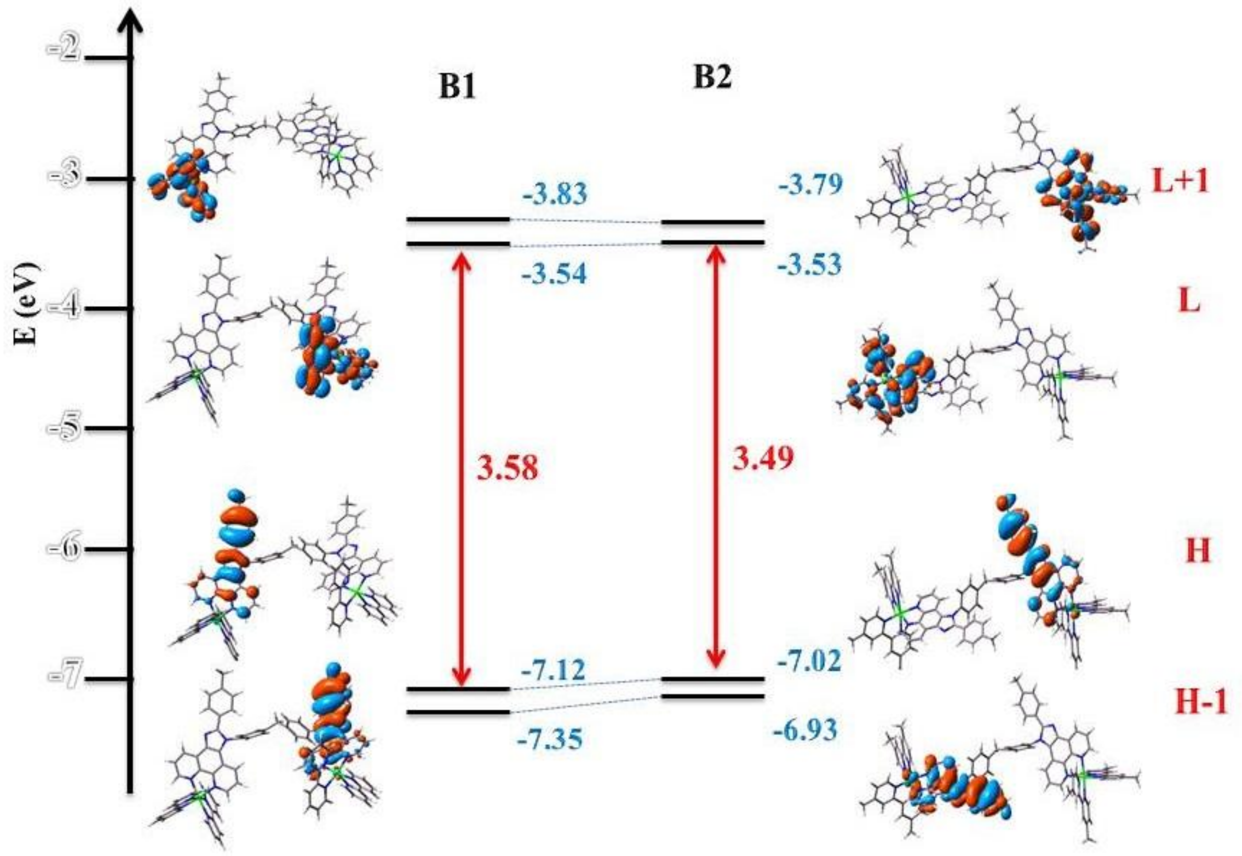


**Figure S5.** Isosurfaces for the HOMO and LUMO of complexes obtained from DFT method through 6-31G* basis set.

**Table S2.** The energy levels complexes B1 and B2 calculated using 6-31G* basis set in the solution phase based on the optimized S0 geometries.

| Complex | HOMO (eV) | LUMO(eV) | Band Gap (eV) |
|---|---|---|---|
| **B1** | -7.12 | -3.54 | 3.58 |
| **B2** | -7.02 | -3.53 | 3.49 |

**Table S3**. EL properties of near infrared light electrochemical cell based on **mononuclear ruthenium** polypyridyl complexes

| **Mononuclear ruthenium complexes** | **Cell configuration** | **EL$_{max}$ (nm)** | $EQE(\%)$ | **Ref.** |
|---|---|---|---|---|
| 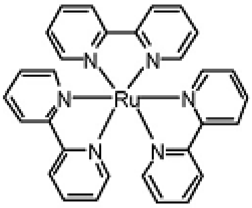 | ITO/complex(90)/ Ga:In | 660 | 1.4 | (7) |
| | ITO/complex (100 nm)/Au (100 nm) | 630 | 0.31 | (8) |
| 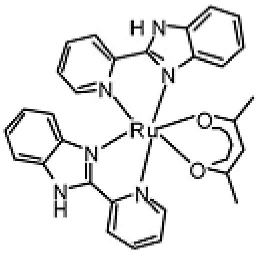 | ITO/complex (100 nm)/Au (100 nm) | 880 | 0.075 | (8) |
| 2 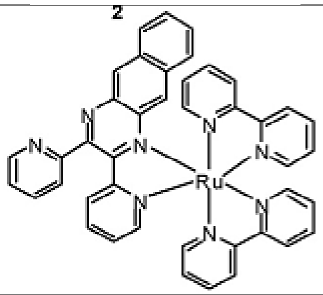 | ITO/complex (100 nm)/Au (100 nm) | 900 | 0.06 | (8) |
| 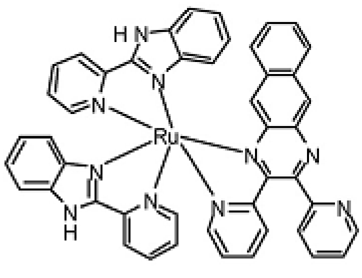 | ITO/complex (100 nm)/Au (100 nm) | 945 | 0.03 | (8) |

| | | | | |
|---|---|---|---|---|
| 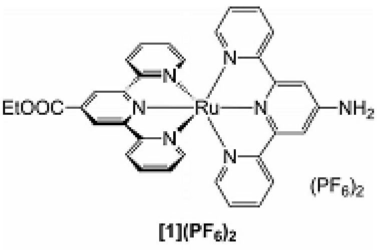 | ITO/PEDOT:PSS(45 nm)/Ru:PMMA (169-194 nm)/Ag | 733 | 0.001 | (9) |
| 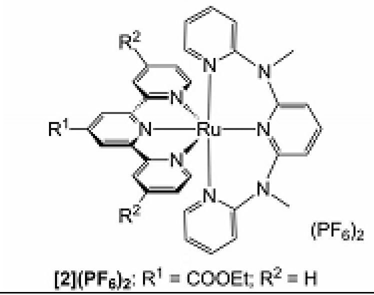 | ITO/PEDOT:PSS(45n m)/Ru:PMMA (169-194 nm)/Ag | 722 | 0.028 | (9) |
| 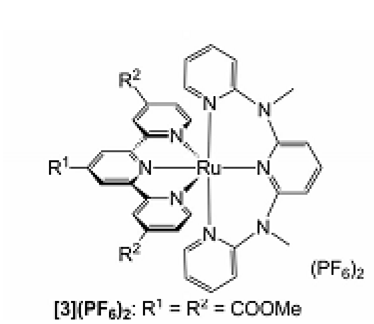 | ITO/PEDOT:PSS(45nm)/ Ru:PMMA (169-194 nm)/Ag | 745 | 0.013 | (9) |
| 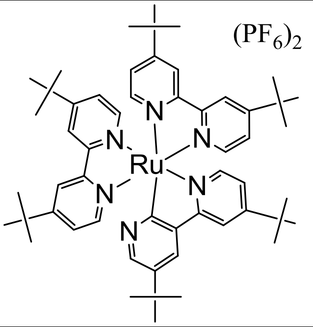 | ITO/PEDOT:PSS(30nm) /Ru/A | 600, 720 | 2.06 ,0.27(afte r 100 min) | (10) |
| 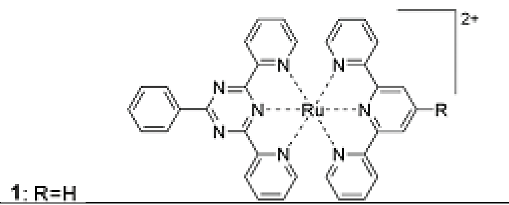 | TO/PEDOT: PSS/ complex:PM [illegible] | 717 | 0.005 | (11) |
| 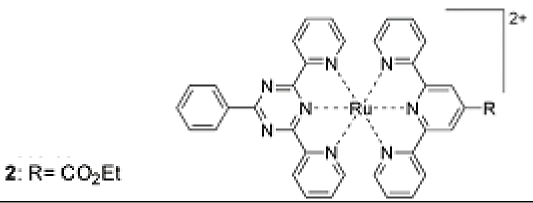 | TO/PEDOT: PSS/ complex:PM [illegible] | 725 | 0.005 | (11) |

| | | | | |
|---|---|---|---|---|
| 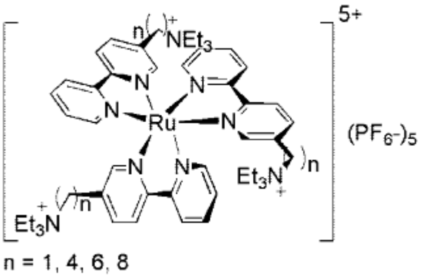 | ITO/[Ru complex]/Au | n=1, 612<br>n=4, 599<br>n=6, 599<br>n=8, 605 | n=1, 0.01<br>n=4, 0.17<br>n=6, 0.43<br>n=8, 0.27 | (12) |

**Table S4** EL properties of light emitting electrochemical cell based on dinuclear ruthenium polypyridyl complexes

| Dinuclear ruthenium complexes | Cell configuration | $EL_{max}$ (nm) | $V_{on}$ (V) | $L_{max}$ (cd.m$^{-2}$) (at voltage) | EQE(%) | Power efficiency (lm/w) | Ref. |
|---|---|---|---|---|---|---|---|
| B1 | ITO/B1, B2 (90nm)/ Ga:In | 635 | 4.5 | 193 (at 7.4) | 0.141 (at 7.5 | - | This work |
| B2 | | 690 | 3.1 | 742 (at 7.7) | 0.682 (at 5.9 V) | - | This work |
| 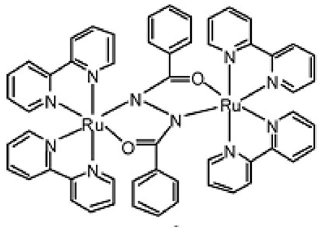 | ITO/complex (100 nm)/Au (100 nm) | 780 | - | - | 0.013 | - | (8) |
| 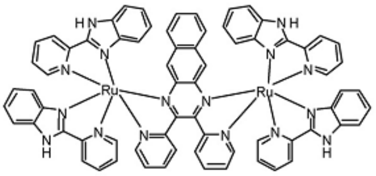 | ITO/complex (100 nm)/Au (100 nm) | 1040 | - | - | - | - | (8) |

| | | | | | | | |
|---|---|---|---|---|---|---|---|
| 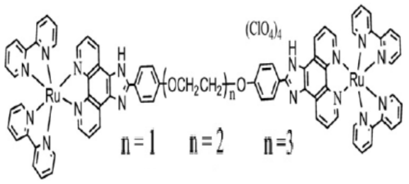 | ITO/complex(90)/Ga:In | n=1, 638<br>n=2, 626<br>n=3, 611 | n=1, 2.3<br>n=2, 2.4<br>n=3, 2.4 | n=1, 310 (5.8V)<br>n=2, 365 (5.8)V<br>n=3, 310 (5.5) V | - | n=1, 0.19 (at 2.7 V)<br>n=2, 0.075 (at 2.7 V)<br>n=3, 0.074 (at 3.3 V) | (13) |
| 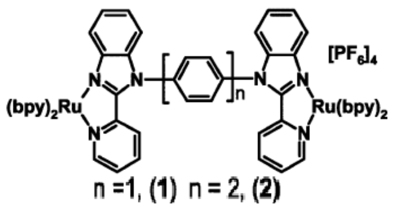 | ITO/complex /Al(100 nm) | n=1, 637<br><br>n=2, 657 | n=1, 3.2<br><br>n=2, 3.5 | n=1, 86 (at 5V)<br><br>n=2, 133 (at 7V) | - | - | (14) |
| 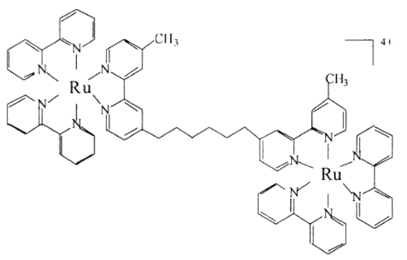 | ITO/ Ru/ Li-triflate /Cr | 638 | 6.8 | - | 0.02 (at 4V) | 0.0014 | (15) |
| 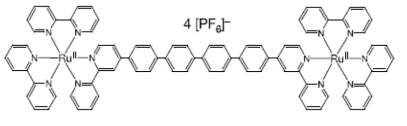 | ITO/Ru:PPV (1:5) /Al(100 nm) | ≈630 (at 4V)<br><br>≈530 (at -4V) | - | 25 (at 4V)<br><br>330 (at -4V) | - | - | (16) |

Table S5. Comparison of lifetime of some polypyridyl Ruthenium complexes in solution and solid phase

| Complexes | sample | τ (ns) | Ref. |
|---|---|---|---|
| B1 | Coated on glass (dry) | 220 | This work |
| B2 | Coated on glass (dry) | 374 ($\lambda_{max}$=690) | This work |
| 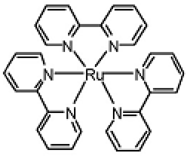 | Acetonitrile solution | 1100 | (12) |
| | water | 358 | (17) |
| | Coated on glass (dry) | 358 | (17) |
| 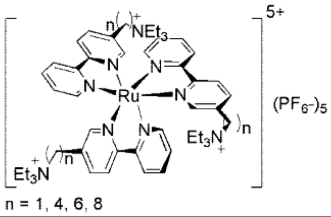 | Acetonitrile solution | n=1, 890 ns<br>n=4, 840 ns<br>n=6, 710 ns<br>n=8, 780 ns | (12) |
| 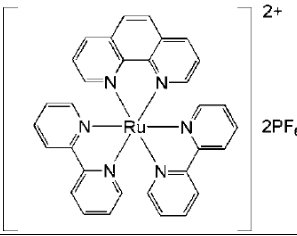 | Acetonitrile solution | 450 | (18) |
| 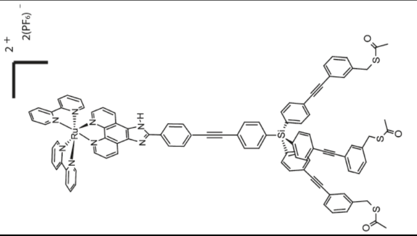 | Acetonitrile solution | 1040 | (19) |
| | Coated on glass (dry) | 900 | |
| 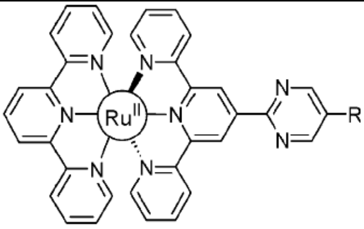 | Acetonitrile solution | R= H, 8 ns<br>R= CN, 200 ns<br>($\lambda_{max}$=675, 713) | (20) |
| 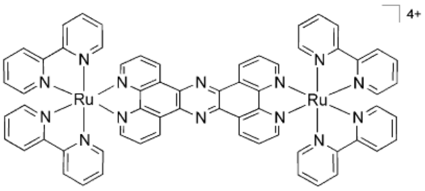 | Cell live | 175 | (21) |

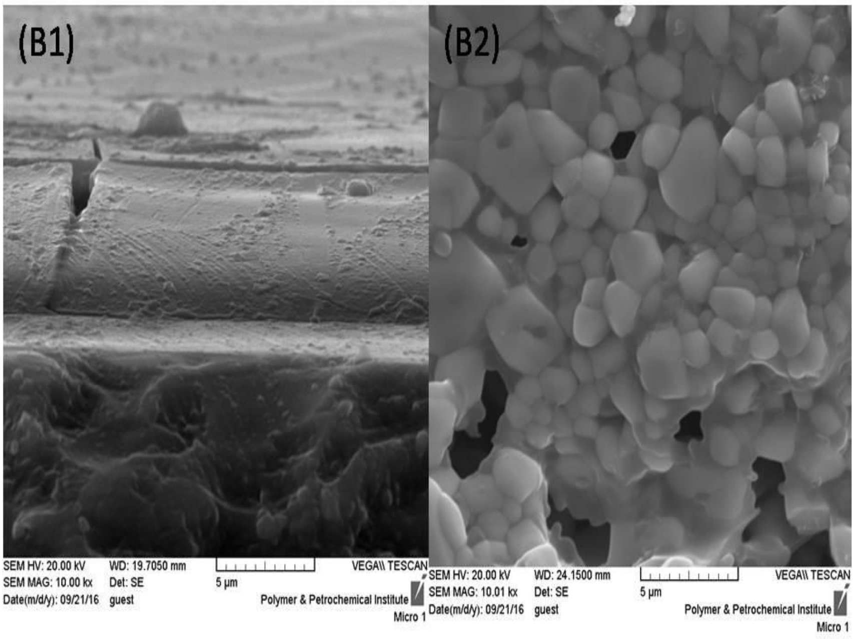


**Figure S6**. Top view SEM images of surfaces of ITO coated with B1 and B2 complexes. The scale bare is 5 micron.

## References:


[1] Yu, Q.Y., Huang, J. F., Shen, Y., Xiao, L.M., Liu, J. M., Kuang, D. B. & Su, C. Y. Novelphenanthroline-based ruthenium complexes for dye-sensitized solar cells: enhancement in performance through fluoro-substitution. RSC. Adv., **3**, 19311-19318 (2013).

[2] Suzuki, K., Kobayashi, A., Kaneko, S., Takehira, K., Yoshihara, T., Ishida, Shiina, H. K., Oishi, S., Tobita, S. Reevaluation of absolute luminescence quantum yields of standard solutions using a spectrometer with an integrating sphere and a back-thinned CCD detector. Phys. Chem. Chem. Phys., **11**, 9850.-9860 (2009).

[3] Sullivan, P. B.;Salmon, D. J.;Meyer, T. Mixed phosphine 2,2'-bipyridine complexes of ruthenium. Inorg. Chem. **17**, 3334-3341(**1978**).

[4] Collin, J. P. & Sauvage, J. P. Synthesis and study of mononuclear ruthenium(II) complexes of sterically hindering diimine chelates. Implications for the catalytic oxidation of water to molecular oxygen. Inorg. Chem., **25**, 135-141(1986).

[5] Caspar, R., Cordier, C., Waern, J. B., Duhayon, C. G., Gruselle, M., Flochm P., Amouri, H. A new family of mono- and dicarboxylic ruthenium complexes $[Ru(DIP)_2(L_2)]^{2+}$ (DIP = 4,7-diphenyl-1,10-phenanthroline): synthesis, solution behavior, and X-ray molecular structure of trans-$[Ru(DIP)_2(MeOH)_2][OTf]_2$. Inorg. Chem. **45**, 4071-4078 (2006).

[6] Paq, W. & Eisenberg, R. Synthesis, characterization, and spectroscopy of dipyridocatecholate complexes of platinum. Inorg. Chem. **36**, 2287-2293 (1997).

[7] Gao, F. G.; Bard, A. J. High-brightness and low-voltage light-emitting devices based on trischelated ruthenium(II) and tris(2,2'-bipyridine) osmium(II) emitter layers and low melting point alloy cathode contacts.Chem. Mater. **14**, 3465-3470 (2002)

[8] S, Xun.; J. Zhang.; X. Li.; D, Ma.; Z, Yuan Wang.; Synthesis and near-infrared luminescent properties of some ruthenium complexes , Syn. Met. **158**, 484–488 (2008).

[9] A, Breivogel.; M, Park.; D, Lee,S, Klassen.; A, Kühnle.; C, Lee.; K, Char.; K, Heinze, Push-pull design of bis(tridentate) ruthenium(II) polypyridine chromophores as deep red light emitters in light-emitting electrochemical cells, Eur. J. Inorg. Chem. 288–295 (2014).

[10] J-H, Hsu, H-C, Su.; Host-only solid-state near-infrared light-emitting electrochemical cells based on interferometric spectral tailoring, Phys. Chem. Chem. Phys., **18**, 5034—5039 (2016).

[11] H, Bolink.;E, Coronado.; R, Costa.; P, Gavina.; E, Ortí.; S, Tatay.; Deep-red-emitting electrochemical cells based on heteroleptic bis-chelated ruthenium(II) complexes, Inorg. Chem. **48**, 3907-3909(2009).

[12] Zysman-Colman, E.: Slinker, J. D.: Parker, J. B.: Malliaras, G.G.: Bernhard, S.: Improved Turn-On Times of Light-Emitting Electrochemical Cells, Chem. Mater. 20, 388 (2008).

[13] Ju, C-C.; Chen, C-H.; Yuan, C-L.; Wang, K-Z. Electroluminescence from single-layer thin-film devices based on three binuclear Ru(II) complexes with different length of flexible bridges. Thin Solid Films, **519**, 3883-3889(2011). [14] Jia, W.-L., Hu, Y.-F., Gao, J. & Wang, S. Linear and star-shaped polynuclear Ru(II) complexes of 2-(2-pyridyl) benzimidazolyl derivatives: syntheses, photophysical properties and red light-emitting devices, Dalton Trans., **0**, 1721–1728 (2006).

[15] Lepretrea, J.-C., Deronziera, A. & Stephan, O. Light-emitting electrochemical cells based on ruthenium(II) using crown ether as solid electrolyte, Synth. Met., **131**, 175–183 (2002).

[16] Welter, S., Brunner, K., Hofstraat, J. W. & De Cola, L. Electroluminescent device with reversible switching between red and green emission. Nature, **421**, 54-57 (2003).

[17] Sciuto, E. L.: Santangelob, M. F.: Villaggio, G.: Sinatra, F.: Bongiorno, C.: Nicotra, G.: Libertinob, S.: Photo-physical characterization of fluorophore $Ru(bpy)_3^{2+}$ for optical biosensing applications, Sensing and Bio-Sensing Research, **6**, 67–71 (2015).

[18] Shaomin Jis, h.: Wu, W.: Wu, W.: Song, P.: Han, K.: Wang, Zh.: Liu, Sh.: Guo, H.: Zhao, J.: Tuning the luminescence lifetimes of ruthenium(II) polypyridine complexes and its application in luminescent oxygen sensing. J. Mater. Chem., **20**, 1953–1963 (2010).

[19] Ramachandra,S.: Schuermann, K. C.: Edafe, F.: Belser, P.: Nijhuis, C. A.: Reus, W. F.: Whitesides, G. M.: Cola, L. D.: Luminescent Ruthenium Tripod Complexes: Properties in Solution and on Conductive Surfaces, Inorg. Chem. **50**, 1581–1591(2011).

[20] Medlycott, E. A.: Hanan, G. S.: Designing tridentate ligands for ruthenium(II) complexes with prolonged room temperature luminescence lifetimes. Chem. Soc. Rev., **34**, 133–142(2005).

[21] Baggaley, E.; Gill, M. R.; Green, N. H.; Turton, D.; Sazanovich, I.V.; Botchway, S. W.; Smythe, C.; Haycock, J.W.; Weinstein, J. A.;Thomas, J. A.; Dinuclear Ruthenium(II) Complexes as Two-Photon, Time-Resolved Emission Microscopy Probes for Cellular DNA, Angew. Chem. Int. Ed. **53**, 3367 –3371(2014).
